# A dose–neutral image quality comparison of different CBCT and CT systems using paranasal sinus imaging protocols and phantoms

**DOI:** 10.1007/s00405-022-07271-4

**Published:** 2022-01-27

**Authors:** Ari-Petteri Ronkainen, Ali Al-Gburi, Timo Liimatainen, Hanna Matikka

**Affiliations:** 1 Department of Clinical Radiology, Kuopio University Hospital, Puijonlaaksontie 2, POB 100, 70029 Kuopio, Finland; 2 Research Unit of Medical Imaging, Physics and Technology, University of Oulu, Oulu, Finland; 3 Department of Diagnostic Radiology, Oulu University Hospital, Oulu, Finland

**Keywords:** Cone-beam computed tomography, Multidetector computed tomography, Paranasal sinuses, Imaging Phantoms

## Abstract

**Purpose:**

To compare the image quality produced by equivalent low-dose and default sinus imaging protocols of a conventional dental cone-beam computed tomography (CBCT) scanner, an extremity CBCT scanner and a clinical multidetector computed tomography (MDCT) scanner.

**Methods:**

Three different phantoms were scanned using dose–neutral ultra-low-dose and low-dose sinus imaging protocols, as well as default sinus protocols of each device. Quantified parameters of image quality included modulation transfer function (MTF) to characterize the spatial response of the imaging system, contrast-to-noise ratio, low contrast visibility, image uniformity and Hounsfield unit accuracy. MTF was calculated using the line spread and edge spread functions (LSF and ESF).

**Results:**

The dental CBCT had superior performance over the extremity CBCT in each studied parameter at similar dose levels. The MDCT had better contrast-to-noise ratio, low contrast visibility and image uniformity than the CBCT scanners. However, the CBCT scanners had better resolution compared to the MDCT. Accuracy of HU values for different materials was on the same level between the dental CBCT and MDCT, but substantially poorer performance was observed with the extremity CBCT.

**Conclusions:**

The studied dental CBCT scanner showed superior performance over the studied extremity CBCT scanner when using dose–neutral imaging protocols. In case a dental CBCT is not available, the given extremity CBCT is still a viable option as it provides the benefit of high resolution over a conventional MDCT.

## Introduction

Although 3D radiographic sinus imaging is not recommended in acute sinusitis, a prolonged or chronic sinusitis may justify a CT scan. Although air-fluid levels and major abnormalities can be observed already in plain sinus radiographs, computed tomography methods provide detailed 3D images of the sinuses, which may help to diagnose sinusitis, to evaluate the state of sinuses, to detect inflammatory diseases and to plan surgical procedures [1]. This applies also to the younger population with higher radiosensitivity [2]. Nowadays, both clinical MDCT scanners and dental CBCT scanners are rather widely available for sinus imaging [3]. Recently also nonsupine extremity CBCT scanners designed for musculoskeletal imaging have been applied for maxillofacial scans with effective radiation doses comparable to a dental CBCT devices and lower than in MDCT [4]. However, the image quality of these options has not been previously been compared objectively nor in a radiation dose–neutral setting.

The commonly reported benefits of CBCT scanners over MDCT are higher resolution with isotropic voxels, lower radiation doses and lower system costs [3, 5–7]. On the other hand, the bulkier, more expensive and versatile MDCTs generally provide a larger field of view, superior signal- and contrast-to-noise ratios and more accurate Hounsfield unit (HU) values [8, 9]. As the scattered radiation limits the image quality of CBCT, it is mainly used for diagnostic imaging of smaller volumes, such as head and neck region or extremities [7, 10]. Although, the fundamental differences between MDCT and CBCT are established, the final clinical performance is determined by the combination of the device performance, the scan protocol, the image reconstruction methods and by the subject to be scanned [11–13]. Nonetheless, studies that would dose neutrally compare the image quality of sinus imaging on MDCT and CBCT scanners are still scarce. Hence, our aim was to compare the image quality produced on equivalent dose levels and on default settings using sinus protocols of an extremity CBCT, a conventional dental CBCT and a clinical contemporary MDCT.

## Materials and methods

The studied CBCT and MDCT scanners included dental CBCT Promax 3D (Planmeca, Helsinki, Finland), extremity CBCT Verity (Planmed, Helsinki, Finland), and a MDCT Somatom Definition Flash (Siemens Healthineers, Erlangen, Germany). Phantom data were acquired using an ultra-low-dose (ULD), a low-dose (LD) and a default (DF) sinus imaging protocols of each device (Table 1). A constant tube current was used in all protocols. Image analyses and quantifications were performed using the open-source software IQWorks (V0.7.2). The study does not include human or animal subjects.

**Table 1:** Imaging protocols for each device

	Dental CBCT			Extremity CBCT			MDCT		
	ULD	LD	DF	ULD	LD	DF	ULD	LD	DF
Tube voltage (kV)	96	96	96	96	96	96	80	100	100
Number of pulses	300	300	400	300	300	400	–	–	–
Tube current (mA)	2.0	2.3	3.6	1.3	3.2	4.0	21	26	64
Exposure time (s)	3	6	12	4.5	4.5	8	0.5	0.5	1
CTDI (mGy)	0.5	1.3	3.7	0.6	1.4	3.9	0.6	1.4	7.0
Voxel size (mm)	0.4	0.4	0.2	0.4	0.4	0.2	0.4 × 0.4 × 0.5		
Scan angle	210	210	210	210	210	210	360	360	360
FOV, height × diameter (mm)	130 × 130 (medium)			160 × 130 (medium)			Collimation 64 × 0.6		

*ULD* ultra-low dose, *LD* low dose, *DF* default, *FoV* field of view

### Phantoms

**Fig. 1:**
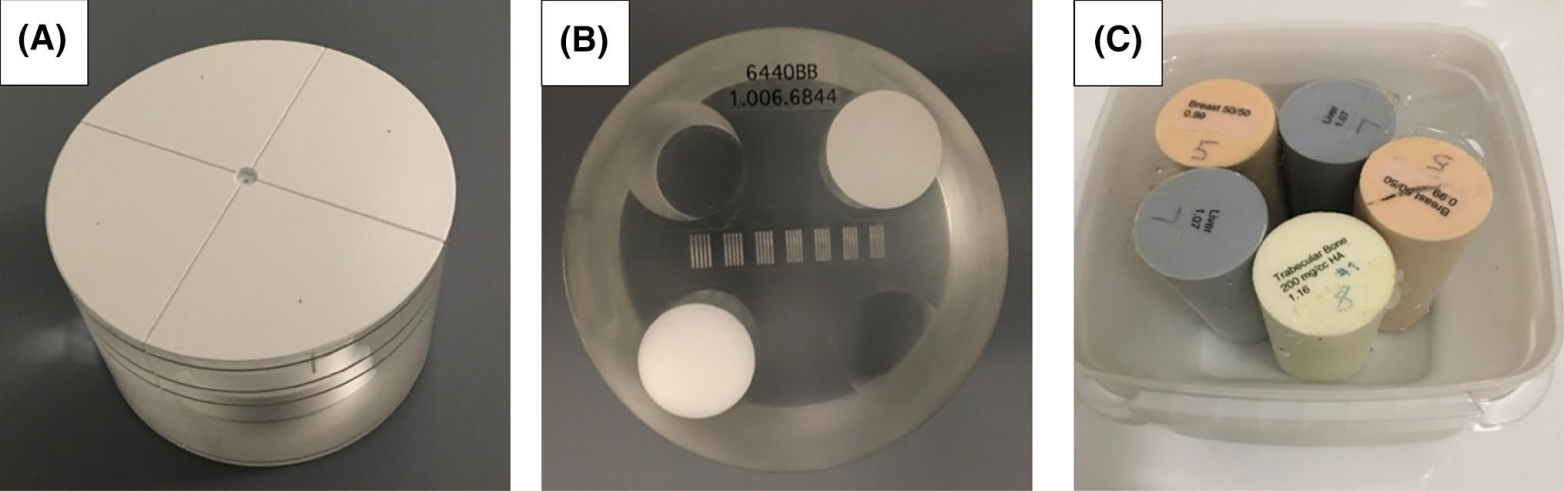
Two commercial cylindrical CBCT phantoms (**A** and **B**) and one custom phantom with commercial electron density rods (**C**) were imaged with all scanners

The image uniformity (homogeneity of HU values) and the image resolution (modulation transfer function, MTF) were measured using a cylindrical CBCT uniformity phantom (Fig. 1A) (diameter 14 cm, height 8.5 cm, part 202078, Instrumentarium Dental, Helsinki, Finland). Another smaller cylindrical CBCT phantom (Fig. 1B) with four inserts of varying attenuation: air (HU = − 1000), polymethyl methacrylate (PMMA, HU = 120), polyvinyl chloride (PVC, HU = − 120) and polytetrafluoroethylene (PTFE, HU = 990), was used to measure the accuracy of HU values (phantom diameter 5 cm, height 7 cm and inserts diameter 1.5 cm, height 3 cm, part number 6440BB, Instrumentarium Dental, Helsinki, Finland). In addition, a third phantom was constructed to quantify the contrast-to-noise ratio (CNR) and the low contrast visibility (LCV) (Fig. 1C). This included two breast tissue rods, two liver tissue rods and one trabecular bone 200 mg/cc hydroxyapatite (HA) rod (electron densities of 0.99, 1.07 and 1.16 g/cc, respectively) of a standardized electron density phantom (Computerized Imaging Reference Systems, Norfolk, VA 23513, USA). The rods (diameter 3 cm, height 5 cm) were firmly placed in a plastic box (width 8.3 cm, height 8.5 cm) and the box was filled with water.

### Image quality metric definitions

**Fig. 2:**
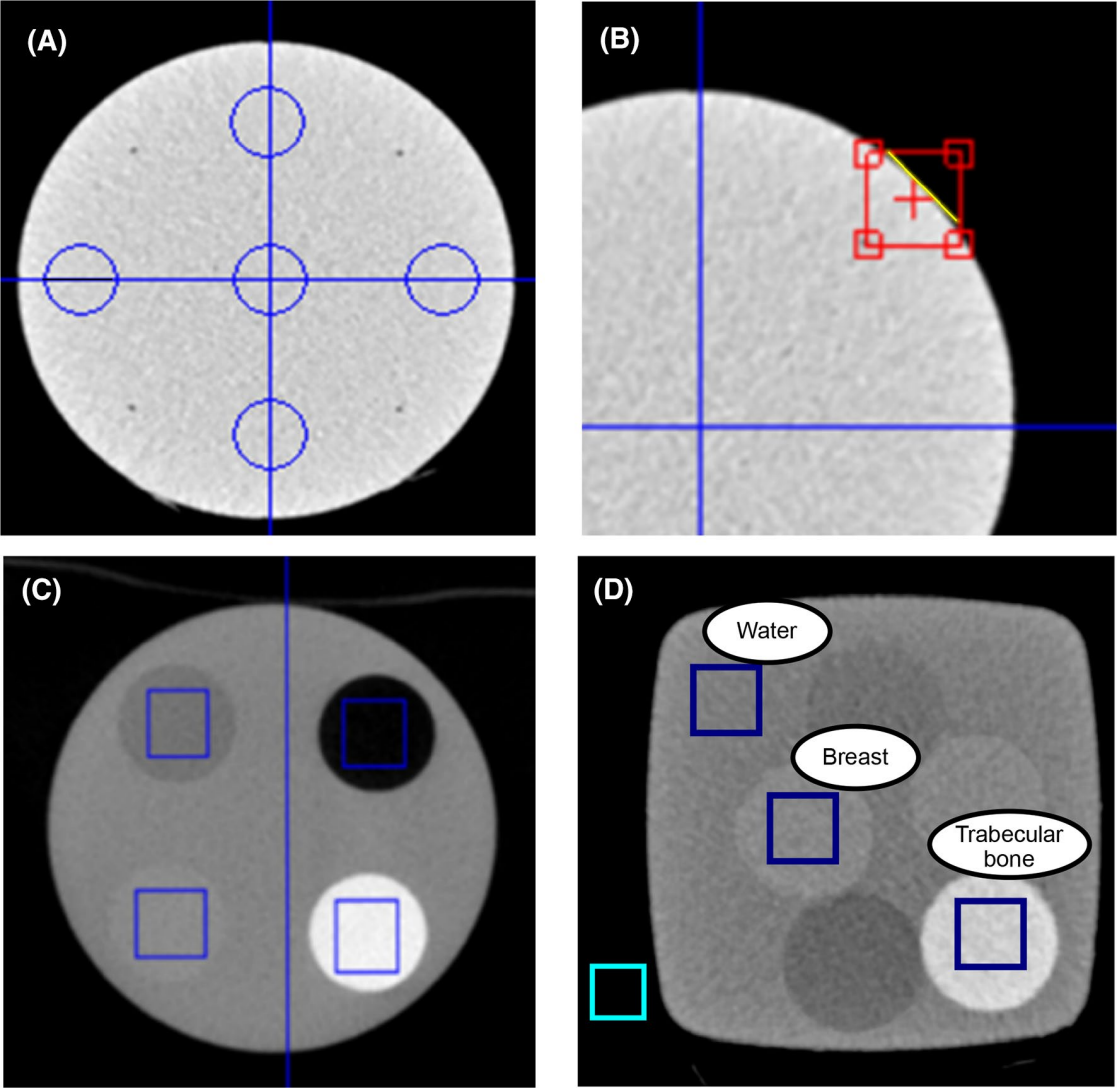
Examples of ROI placement in the phantom measurements. **A** Uniformity phantom and the five ROIs used in the calculations. **B** Edge detection ROI for ESF measurement and MTF calculation. **C** Representative axial slice from the HU-value phantom with ROIs. **D** Breast and trabecular bone rod ROIs for LCV calculations, and water, trabecular bone, and background ROIs for CNR measurements

The uniformity was calculated from one central region of interest (ROI) and four peripheral ROIs (Fig. 2A) as the mean difference between the central ROI and the peripheral ROIs (Eq. 1)

1 $$\begin{array}{*{20}c} {{\text{Uniformity}} = \frac{1}{4}\mathop \sum \limits_{i = 1}^{4} (m_{c} - m_{p,i} ) } \\ \end{array}$$

Here ${m}_{\mathrm{c}}$ is the mean HU value from the central ROI and ${m}_{\mathrm{p},i}$ is the mean value from *i*^th^ peripheral ROI (3, 6, 9 and 12 o’clock locations with 1 cm diameter). The modulation transfer function (MTF), which describes how well spatial frequency information is transferred through the imaging system, was calculated from a measured edge spread function (ESF) close to the scanner isocenter [14]. To achieve MTF, an edge is first identified (Fig. 2B) and an ESF is sampled. Then the ESF is differentiated to yield a line spread function (LSF)

2 $$\begin{array}{*{20}c} {LSF\left( x \right) = \frac{d}{dx}ESF\left( x \right)} \\ \end{array}$$

Finally, from the LSF a normalized MTF can be calculated [15]

3 $$\begin{array}{*{20}c} {MTF\left( f \right) = \left| {\frac{{FT\left\{ {LSF\left( x \right)} \right\}}}{{\mathop \smallint \nolimits_{ - \infty }^{\infty } LSF\left( x \right)dx}}} \right|} \\ \end{array}$$

where $FT\left\{ \cdot \right\}$ indicates Fourier transform. The integral normalizes MTF to unity (all information transferred) at the zero frequency. The accuracy of HU values was evaluated by calculating the mean HU values from rectangular ROIs (Fig. 2C) and comparing these to the known HU values. The low contrast visibility (LCV) was defined as

4 $$\begin{array}{*{20}c} {LCV = 2\frac{{|m_{{\text{w}}} - m_{{{\text{br}}}} |}}{{\sigma_{{\text{w}}} + \sigma_{{{\text{br}}}} }}} \\ \end{array}$$

where ${m}_{\mathrm{w}}$ and ${m}_{\mathrm{br}}$ are the mean HU values, and ${\sigma }_{\mathrm{w}}$ and ${\sigma }_{\mathrm{br}}$ are the standard deviations in ROIs over the water (w) and breast (br) insert, respectively. Furthermore, CNR was measured as

$$CNR = \frac{{|m_{tb} - m_{w} |}}{{\sigma_{bg} }}$$

where ${m}_{\mathrm{w}}$ is the mean HU value in the water ROI, and ${\sigma }_{\mathrm{bg}}$ is the standard deviation in the background ROI. To take into account, the radiation dose in comparisons, we also calculated the dose normalized versions of CNR (CNRD) by dividing CNRs with $\sqrt {{\text{CTDI}}_{{{\text{vol}}}} }$ [16–18].

## Results

Overall, the dose–neutral comparisons show that the MDCT had better uniformity and contrast metrics (CNR, CNRD and LCV) compared to the CBCT scanners (Table 2), while CBCT scanners had superior resolution (MTFs) compared to the MDCT. On the accuracy of HU values, the MDCT and the dental CBCT outperformed the accuracy of the extremity CBCT. Interestingly, the dental CBCT showed superior performance with dose-matched protocols over extremity CBCT in all the studied objective metrics (Table 2).

**Table 2:** Calculated image quality metrics for dose–neutral low dose imaging protocols and for default sinus imaging protocols for each device

Scan protocol	Ultra-low dose			Low dose			Default		
Scanner	Dental CBCT	Extremity CBCT	MDCT	Dental CBCT	Extremity CBCT	MDCT	Dental CBCT	Extremity CBCT	MDCT
CTDI (mGy)	0.5	0.6	0.5	1.3	1.4	1.4	3.7	3.9	7
Uniformity (HU)	67	125	7	56	100	6	51	88	6
CNR	7.2	4.5	19.1	18	11.4	26.4	26	12.2	40.4
CNRD	10.2	5.8	25.3	15.9	9.6	22	13.5	9.8	15.2
LCV	0.78	0.48	1.14	1.43	1.18	2.03	2.51	1.20	3.41
Air (HU)	− 964.6	− 971.3	− 1022.9	− 986.8	− 959.2	− 1023.7	− 999.3	− 973.6	− 1022.8
PTFE (HU)	984.8	1429.5	1149.5	968.7	1377.3	1155.4	940.6	829.5	1113.9
PMMA (HU)	154.5	29.9	134.1	118.1	89.9	138.7	93.7	61.4	151.7
PVC (HU)	− 88.7	9.6	− 123.6	− 114.2	− 129.8	− 122.1	− 137.5	− 155.3	− 94.6
Air error%	3.5%	2.9%	2.3%	1.3%	4.1%	2.4%	0.1%	2.6%	2.3%
PTFE error%	0.6%	44.4%	16.1%	2.2%	39.1%	16.7%	5.0%	16.2%	12.5%
PMMA error%	28.7%	75.1%	11.8%	1.6%	25.0%	15.6%	26.3%	48.8%	26.4%
PVC error%	26.1%	108.0%	2.6%	4.9%	8.1%	1.8%	14.6%	29.4%	21.2%
MTF 10% (lp/mm)	1.33	0.93	0.52	1.34	0.89	0.53	1.46	1.00	0.48
MTF 50% (lp/mm)	0.65	0.46	0.27	0.66	0.48	0.29	0.69	0.40	0.28

### Image uniformity

**Fig. 3:**
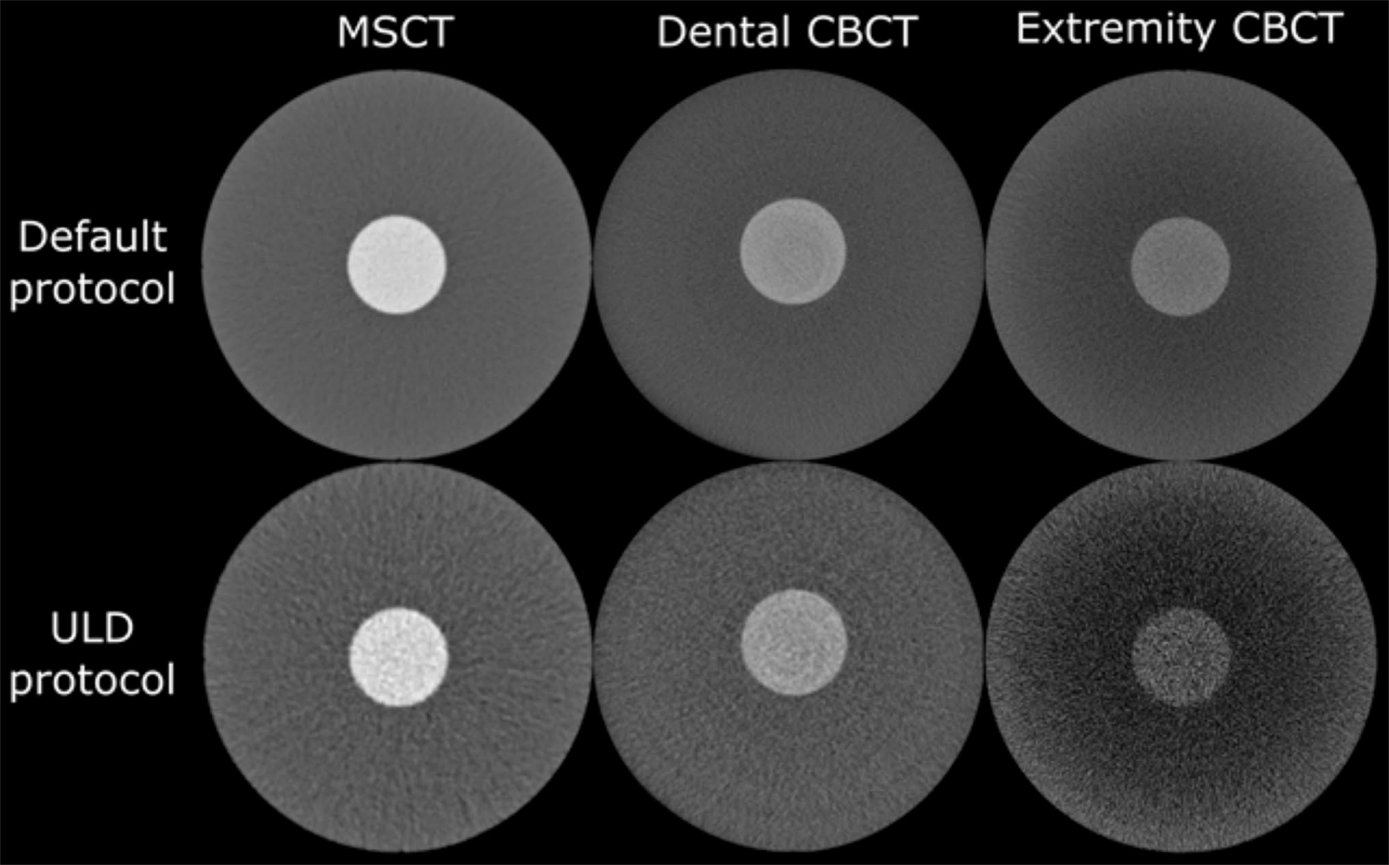
Example axial images of the uniformity phantom acquired with the default and ULD protocols for each scanner

The MDCT produced the best HU uniformity at all dose levels, as the mean difference between the peripheral ROIs and the central ROI differed less than 10 HUs. Dental CBCT protocols produced differences in the range 50–60 HUs, whereas extremity CBCT had the worst uniformity, producing mean differences in the range 80–120 HUs. These differences in uniformity can also be visually appreciated (Fig. 3).

### Resolution

**Fig. 4:**
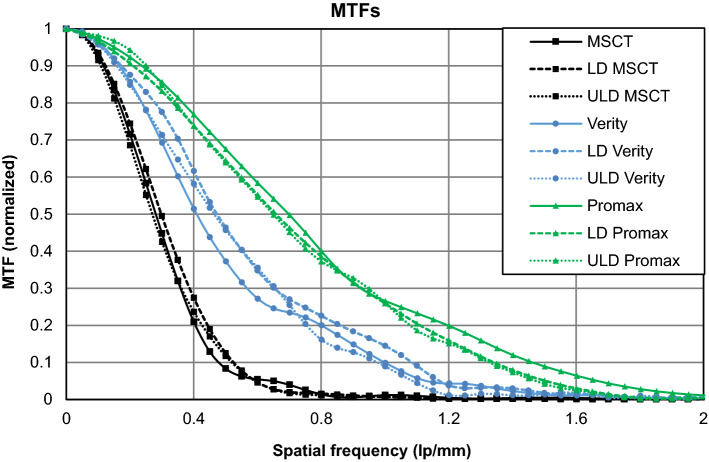
Modulation transfer functions for each scanner at different dose levels

Based on the MTF curves (or MTF50 and MTF10 values) the dental CBCT had the highest resolution among the devices followed by the extremity CBCT. The conventional MDCT clearly had the lowest MTF. The MTF curves within a single device did not vary substantially between the dose levels (Fig. 4).

### Low contrast visibility

**Fig. 5:**
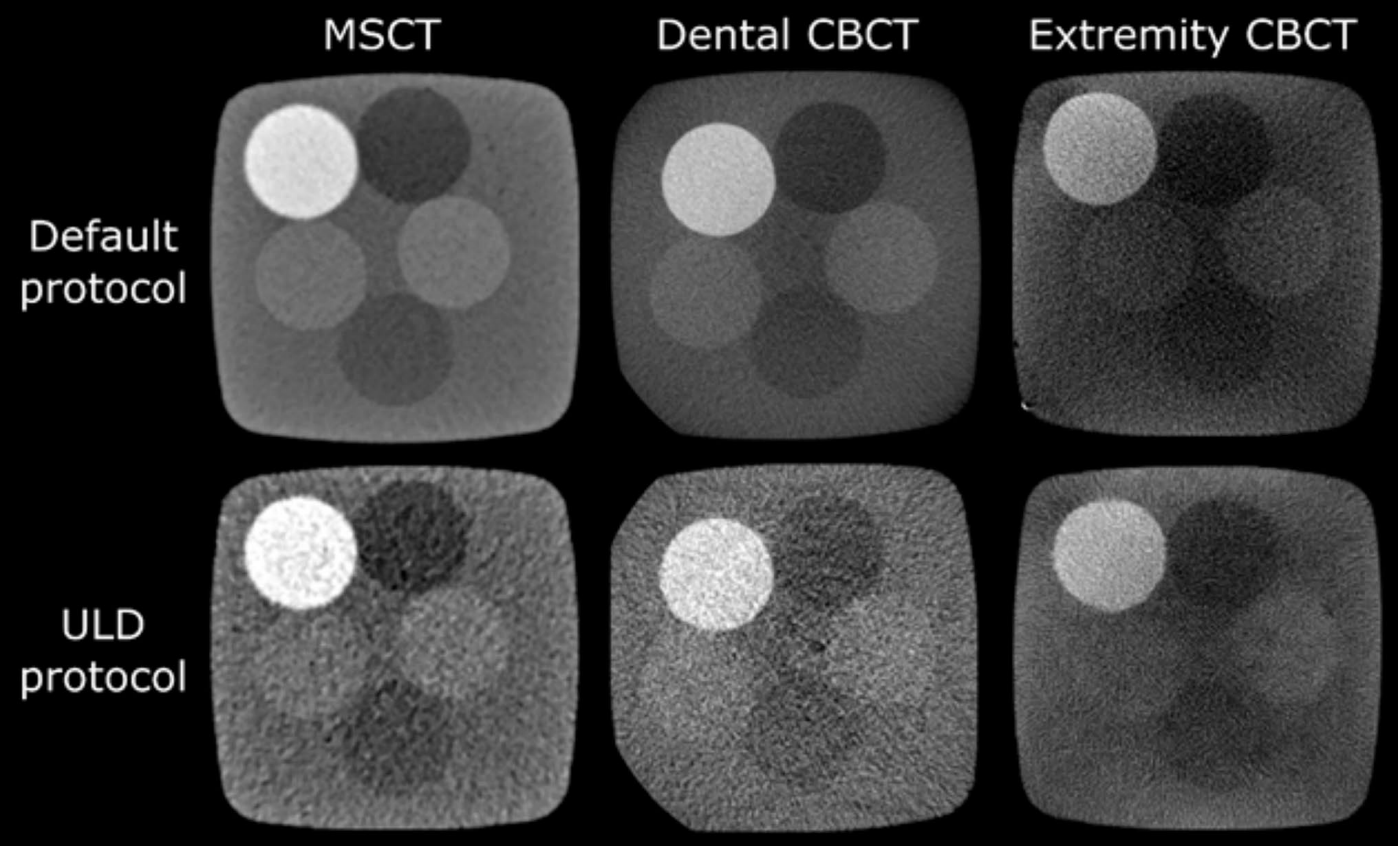
Example axial images of the low contrast phantom acquired with the default and ULD protocols for each scanner

The extremity CBCT had the lowest LCV between the devices (LCV values of 0.48, 1.18 and 1.20 for ULD, LD and DF protocols, respectively). Comparing these to the values of dental CBCT (0.78, 1.43 and 2.51) and MDCT (1.14, 2.03 and 3.41) shows that the LD protocols of the other two devices had better LCV than the default (DF) protocol of the extremity CBCT. An explaining factor could be the visually appreciated cupping artefact in the extremity CBCT images (Fig. 5). The MDCT performed better than dental CBCT when similar protocols are compared head-to-head.

## Discussion

In clinics that have several comparable devices for scanning the same region, an objective and dose neutral assessment of image quality helps in choosing the primary method. In this study we aimed to compare the performance of the routinely used dental CBCT to an extremity CBCT and contemporary MDCT scanner in sinus imaging using protocols of equivalent radiation doses. The CBCT scanners had clearly superior image resolution compared to the MDCT, as seen qualitatively from the images and objectively from the MTFs. Thus, small findings with good inherent contrast, such as bony structures, are better visualized in CBCT images. However, the conventional MDCT provided superior contrast-to-noise ratio, low contrast visibility and image uniformity compared to the two CBCT scanners at similar dose levels. Hence, the MDCT should perform better in detection and characterization of findings with minor HU alterations. A bit surprisingly, the dose-matched sinus protocols of the extremity CBCT provided inferior image quality in all studied aspects as compared to the dental CBCT scanner. As such, the studied dental CBCT scanner should be preferred over the studied extremity CBCT scanner for sinus imaging.

The studied image quality properties differ between the machines because of variations in imaging geometry, radiation beam properties, detector technology and postprocessing algorithms. When comparing the studied CBCT devices, the dental CBCT unit has slightly longer source-to-image distance (600 mm as compared to 580 mm) and the detector rotates closer to the head during imaging than in the extremity CBCT, which result in sharper projection images with less penumbra [19]. This partly explains the superior image quality of the dental CBCT. The same flat panel detectors are used in both studied CBCTs (127 µm pixels with an active surface area of 302 × 249 mm), as the devices are produced by the same manufacturer. When comparing the MDCT and CBCTs, the most important differences are in beam shape, detector assembly and reconstruction algorithms. The large cone angle of the CBCT radiation beam results in high amount of scatter that adds random noise to the final images. Further amplification of image noise occurs when small voxels that are available in CBCT due to the flat-panel detector technology are used (even sub 0.1 mm^3^ voxels). These differences evidently lead to the observed inferior homogeneity, and lower CNR and LCV values of the CBCTs compared to the MDCT scanner. However, both extremity and dental CBCT are likely better suited for routine sinus imaging due to their higher resolution. In future, novel reconstruction methods, such as those based on deep learning [20], may significantly improve the scatter correction and reduce image noise and uniformity of the CBCT scanners.

### Limitations

We used phantoms to compare image quality of sinus imaging protocols that had identical or similar dose level according to the CTDI_vol_. The phantoms were not a good representation of human geometry, as they were not anthropomorphic. However, there is no reason why the overall performance quantified with our phantoms would not correlate to clinical image quality. Secondly, it is noteworthy that the CTDI does not directly present the effective dose of the patient, which is likely a bit lower in the CBCT compared to MDCT scanners due to their different scan geometry [4]. Lastly, the current results are valid for the studied CBCT and MDCT scanner models with the used sinus imaging protocols and reconstruction settings, and as such, drawing conclusions to a broader device spectrum must be done with caution. In addition, the study does not include the newest and emerging technologies, such as supine CBCT scanners with even better resolution or latest high-resolution bone imaging modes of MDCT, which has been reported to provide results similar to CBCT, at least when the aspect of radiation dose is ignored [21]. Another interesting new technology is tin-filtered ultra-low-dose MDCT imaging, which reportedly allows sinus imaging with a very low effective dose (at level 0.01–0.02 mSv) [22]. Moreover, artificial intelligence, or strictly speaking, deep learning reconstruction algorithms that are slowly emerging were not considered here. These may prove to be extremely useful in future for enhancing the image quality through reduced image noise or image artefacts in both CBCT and MDCT images [23].

### Conclusions

The dedicated extremity CBCT (nonsupine) was inferior to the dental CBCT device, but offered better spatial resolution compared to the MDCT. This makes it a viable option for sinus imaging in cases where dental CBCT is not available and high resolution is warranted (e.g. small fractures or bony changes). On the other hand, the images produced by MDCT had better contrast and uniformity compared to the CBCT scanners at equivalent radiation dose. Hence, sinus imaging with MDCT could be preferred in cases where the soft tissue delineation is desired, e.g. in postoperative imaging, particularly when MRI is not feasible.
